# Bugbears in the Waiting Room: Revisiting Arber and Sawyer's Classic Study of GP Reception Work Using Ethnography in Eight English General Practices

**DOI:** 10.1111/1467-9566.70104

**Published:** 2025-10-13

**Authors:** Catherine Pope, Annelieke Driessen, Abi Eccles, Bella Wheeler, Carol Bryce, Jacob Heath, Chloe Phillips, Toto Gronlund, Helen Atherton

**Affiliations:** ^1^ Nuffield Department of Primary Care Health Sciences University of Oxford Oxford UK; ^2^ Department of Anthropology University of Amsterdam Amsterdam the Netherlands; ^3^ Unit of Academic Primary Care Warwick Medical School Coventry UK; ^4^ Public Representative. C/O Nuffield Department of Primary Care Health Sciences University of Oxford Oxford UK; ^5^ School of Primary Care Population Health and Medical Education University of Southampton Southampton UK

**Keywords:** access, ethnography, general practice, receptionists

## Abstract

In 1985, Arber and Sawyer described the discretionary rationing power of general practice receptionists. Our paper revisits this territory. Much has changed in the intervening decades. Digitalisation has altered reception work. Increasing multimorbidity and rising chronic illness, combined with a dwindling workforce, restricted funding and systemic pressures on public services, have fuelled the ‘crisis’ in general practice. ‘Unacceptable’ delays in getting a general practitioner (GP) appointment are seen as evidence of this. Our focused ethnography in eight English NHS general practices highlights important shifts in receptionists' management of GP access. We observed waiting and reception areas, interviewed 70 staff and 74 patients and examined practice documents. Arber and Sawyer's dragon metaphor remains salient, but receptionists have new strategies of bureaucratic distancing and redirection to manage appointment requests. They are gatekeepers still and remain a target for hostility but mitigate this by using these strategies. Patients on a quest to obtain an appointment with a GP may still be thwarted and sometimes meet dragons at the desk, but they may more often find themselves allied with the receptionist. The barrier to getting a GP appointment has become the access system and its discorporate digital forms, rather than the receptionist or the GPs she protects.

## 1 Introduction

This paper is about access to healthcare. It examines how receptionists work as gatekeepers to general practitioners (GPs, otherwise known as family doctors) in the UK National Health Service (NHS). It was, in part, inspired by a classic sociological paper by Arber and Sawyer (1985), which characterised these administrative staff as ‘dragons behind the desk’.

Arber and Sawyer's papers (1981, 1985) noted that the almost continual reconfiguration of the UK National Health Service (NHS) since its introduction in 1948 had increased its complexity and reduced accessibility. They argued that the GP receptionist had become central to the problem of access to primary healthcare. Their arguments were well made and sat alongside a wider sociological literature about ‘lower order participants’ (Mechanic 1962) or ‘street level bureaucrats’ (Lipsky 1980) who exercised power and influence in organisations. People processing work (Prottas 1978, 1979; Keiser 2010) and the ways in which these kinds of staff exercise discretion and determine eligibility and access to services continue to be of interest to medical sociologists.

Arber and Sawyer used a 1977 interview survey of a representative sample of 1000 adults from London and the South East to explore patients' views of general practice. They reported that one third of patients found it difficult or impossible to get a same‐day appointment and 40% had negative experiences when communicating with receptionists. The receptionists' gatekeeping role fuelled considerable public hostility and led to the portrayal of GP receptionists as ‘battle axes’ and ‘dragons’. Patients were, they suggested, becoming more antagonistic towards receptionists, who were perceived as officious, interfering and intrusive. Arber and Sawyer concluded that the increasing complexity of general practice, coupled with drives for organisational efficiency, would only encourage more rationing of requests for appointments, which, in turn, would encourage more public hostility.

More recent contributions to general practice research have continued to explore the intermediary and gatekeeping roles of reception staff. Brant et al. (2018) showed that reception staff continue to use initiative to manage demand for appointments, and Litchfield et al. (2023) noted the increased visibility of the reception role and its impact on patient perceptions of primary care. The portrayal of receptionists as dragons has also been re‐examined in medical journals: For example, Hammond et al. (2013) showed that receptionist power was nuanced and contingent, and Grant et al. (2016) elaborated on the articulation work performed by receptionists, notably in relation to managing repeat prescribing. Research has also provided insights about receptionists' feelings of accountability to patients (Swinglehurst et al. 2011), and Ward and McMurray (2011) revealed that these frontline staff had to develop strategies for emotion management in their role. Most recently, Checkland et al. (2025), noting the increasing challenges associated with the reception role, called for further research about these essential but overlooked healthcare staff.

It is 40 years since Arber and Sawyer published their research and, perhaps, timely to revisit their ideas about dragons and the hostility felt by patients and the public towards reception staff. Our paper explores how reception staff manage access to GP appointments, seeking to understand if, and how, this role has changed and whether the hostility towards receptionists has increased as Arber and Sawyer predicted. We seek to contribute both to the sociological literature on lower‐order participants and patient–receptionist interactions and to the wider understanding of access to healthcare. We also examine the significant changes to general practice, notably the digitalisation of the bureaucratic work associated with access to the GP.

Before describing our empirical field and the methods used, the next section considers contemporary general practice and the problem of access, highlighting important changes to reception work since Arber and Sawyer's paper was published.

## 2 General Practice and the Problem of Access

UK NHS primary healthcare is a public service provided via a system of independent, small‐ to medium‐sized businesses owned by GP partnerships. GPs offer generalist medical services similar to primary care physician or family doctor services found in other countries; approximately 90% of all NHS patient contacts are with general practice (McCarthy and BMA 2024). Practices oversee a list of registered patients; the size of these lists has grown (RCGP 2024) as larger practices and the consolidation of multiple partnerships, some sharing premises and/or administrative staff, have become widespread. The vast majority of contacts with general practice are handled initially by receptionists who are now sometimes referred to as patient liaison or patient services staff.

Arber and Sawyer described the difficulties patients encountered in dealing with GP receptionists in the late 1970s. The access problems they described persist. In the run‐up to the 2024 UK general election, news media were replete with headlines such as ‘Patients told to queue for an hour to get an appointment’ (Tait 2024) and ‘One in 20 patients wait at least 4 weeks for an appointment with their GP’ (D. Campbell 2024). The inaccessibility of general practice has been frequently documented by UK think tanks and independent research (Baird et al. 2016; Wensing et al. 1998). GP access has been a persistent bugbear (source of annoyance) for successive UK governments, but this access problem is not unique to the UK. A recent narrative review highlighted an average wait of 4 days to access primary care in Australia, the UK and Germany and reported a Canadian study that showed wait times of more than 2 weeks (McIntyre and Chow 2020). However, in the UK, the problem of GP access appears especially salient as a key indicator of a wider crisis in NHS primary care. This crisis is also characterised by a dramatic decline in GP workforce numbers, the collapse of the partnership model of practice ownership, crumbling practice buildings and practice closures, all set against a backdrop of an increasingly multimorbid and ageing population demanding ever more healthcare (British Medical Association 2024). General practice has been described as ‘unmanageable’ (Croxson et al. 2017) and ‘unviable’ (Mathew 2021), and high GP workloads are associated with stress and burnout (RCGP 2022).

To address these issues, in recent years new primary healthcare professional roles have been introduced to work alongside GPs, including physician assistants, paramedics, physiotherapists and advanced nurse practitioners (NHS England 2023). Nonclinically trained staff, such as social prescribers (who provide care navigation support and self‐management advice), have also been employed, in addition to more clerical and administrative staff. Outside the setting of general practice, attempts have been made to repurpose community pharmacists as a source of generalist health advice, notably via the Pharmacy First scheme (NHS England, undated), and public health education has tried to encourage patients to undertake more self‐care rather than seeking appointments with their GP. GPs are seeing more patients than ever before (NHS England 2025), but patients still struggle to get appointments (Wise 2024).

### 2.1 Changes to GP Reception Work

Over the years, numerous political and policy interventions have been tried to reorganise and improve the way patients get GP appointments (Eccles et al. 2024). The move away from unmanaged in‐person waiting rooms to formal appointment systems in the 1960s was initially welcomed by GPs (Moore 2022), and receptionists were crucial to the administration of these systems, interacting with patients to manage requests for appointments. One of the most profound changes to general practice, and to reception work, has been the introduction of digital information and communication technologies. Where once the receptionist solely interacted in person with the patient at the reception desk or by telephone, she (and it is still likely that the receptionist is female (Litchfield et al. 2022)) now deals with electronic health records, digital telephony, email communications and online web‐based services, including digital triage/assessment and booking. Other tasks, such as health screening and medicines prescribing, have also been digitalised and semi‐automated thanks to computerisation. As a result, many administrative tasks no longer take place at the reception desk in the practice waiting area visible to patients but are instead performed in back offices away from the public gaze. Reception staff increasingly work in large hierarchical teams, alongside practice managers and other staff employed to undertake the digital work associated with modern general practice. The use of digital systems for making appointments and delivering GP consultations remotely expanded further in 2020–2021 in response to the COVID‐19 pandemic. General practice is now expected to provide increased digital access while also returning to offering face‐to‐face appointments.

Receptionists are in the front line of managing demand for GP appointments, and their work has been at the forefront of digitalisation. This paper reports the analysis from eight ethnographic case studies exploring GP access and the gatekeeping work of receptionists, including how digitalisation has impacted on receptionist–patient encounters. The next section describes the study and methods in more detail.

## 3 The Study

As part of a larger study of GP access funded by the National Institute for Health and Care Research (NIHR), we conducted observational fieldwork and semi‐structured interviews with staff and patients at eight English general practices (see Table 1). The practices were purposively identified prior to fieldwork using a schematic representation of access systems informed by a scoping review of evaluations of GP access systems (Eccles et al. 2024). The practice sites varied by level of deprivation (Index of Multiple Deprivation score), location (rural/urban), number of GPs and list size.

**TABLE 1:** List size (number of registered patients), deprivation score, location and previously used access system for our 8 case study sites.

Site ID	List size	IMD^a^	Location	Previously used access system
A	33,000	8	South, coastal/market town	Advanced access—A system that encourages immediate or same‐day appointment booking
B	13,000	9	Midlands, rural/village	Telephone first—All patients are contacted by a GP on the phone before scheduling an appointment.
C	8000	5	North‐east, urban/commercial	Telephone first
D	5,000	6	Midlands, suburban	Telephone triage by a GP or nurse to assess a patient's health concerns before scheduling an appointment
E	20,000	10	South‐east, market town	Alternative consultation (the use of email, video and phone consultations, often in conjunction with an online e‐consultation assessment prior to scheduling an appointment)
F	9000	1	North‐west, urban/ex‐industrial	Telephone first
G	12,000	3	North, suburban/ex‐council (social) housing	N/A (sampled for geography/deprivation)
H	12,000	3	North, urban/ex‐industrial	N/A (sampled for geography/deprivation)

^a^This considers various factors such as income, employment, education, health and living environment and ranks areas on a 10‐point scale. 10 denotes a geographical area that is in the least deprived decile, and 1 denotes the most deprived. However, this may mask local variations; for example, one of the less deprived sites served a hotel that housed refugees and asylum seekers.

Abbreviation: IMD = Index of Multiple Deprivation score.

Six of the selected practices had previously been the subject of research about their access systems. The systems previously used included ‘Advanced Access’, which emphasises meeting demand on the day (Salisbury, Montgomery, et al. 2007; Salisbury, Goodall, et al. 2007); ‘Alt‐Con’, which offers patient consultations via email, video call and telephone (Atherton et al. 2018); and variants of ‘telephone triage’ and ‘telephone first’, which use telephone screening and prioritisation prior to offering an appointment (J. L. Campbell et al. 2014; Newbould et al. 2017, 2019). Two additional practices were sampled to represent geographical areas and populations with higher levels of socioeconomic deprivation. Although these two practices had not been formally involved in previous access system evaluations, they had used different access modalities, notably in the pandemic, where online and telephone access dominated. One was also making greater use of the Additional Roles Reimbursement Scheme (ARRS) staff (NHS England 2023) to employ other care providers who were not GPs. Each general practice was approached with the assistance of local NIHR Clinical Research Networks.

### 3.1 Data Collection

The research team collected data between November 2022 and February 2024, spending between four and eight weeks in each general practice. Data collection began with familiarisation with the assigned practice and was followed by targeted observations. Posters were displayed to inform people when observations were being conducted, and staff members, patients and those accompanying patients were able to decline being observed by alerting staff or the researcher. We collected documents pertaining to the access system (e.g., protocols, receptionist training materials and posters) to inform our understanding. We made handwritten field notes contemporaneously or made these records as soon as possible after observation/conversations. We observed office areas, reception desks, waiting rooms and relevant practice meetings. Observational data collection included informal conversations with staff and patients and allowed us to capture not only verbal but also nonverbal expressions. We frequently drew on these notes in later interviews or informal conversations.

We interviewed staff and patients about the practice access system and how access to appointments was managed. Patient interviews explored their interactions with the practice, how they made appointments and their view of changes or developments to access systems and the impacts of these. Staff interviews explored their experiences and the decision‐making process surrounding the implementation of access systems and changes to access over time.

Interviews were semi‐structured; interviewers used a topic guide but also explicitly drew on ethnographic observation to probe deeper into specific observations, words and phrasings we had heard repeatedly, and/or seeming contradictions between what was said and what was done. We interviewed 74 patients (45 self‐identified as women, 29 as men) aged between 18 and 80 years, 46 of whom declared a long‐term condition or disability. Not all patient interviewees provided demographic information, but 12 people self‐identified as Arab, Asian, Black, Indian or Pakistani. We also interviewed 70 practice staff. Interviews were arranged at a mutually convenient time and took place in a private office if face to face or by telephone or videoconferencing platform. Staff were invited to interviews by email from the practice manager or GP, and the practice (usually via a practice manager) was asked to invite potential patients for interview in accordance with our sampling frame (which sought to include variation in age, ethnicity, gender and presence/absence of long‐term conditions or disabilities). Invites were sent by email, letter, text message or given in person. Staff interviews included GP partners, salaried GPs and GP trainees and locums, practice nurses and other allied health roles, and different administrative staff who might include practice managers, receptionists, patient care or clinical coordinators, patient services staff and, at one practice, a refugee care coordinator. Interview questions and topics were trialled with members of the study's patient and public involvement panel.

Being physically present in the practice allowed the researchers to ‘get a feel’ for each practice site and to directly observe what receptionists and patients did in addition to the interview accounts, where they described what they did and how they felt. This resulted in much ‘thicker’ data than could be collected from an interview study alone. The two methods were iteratively combined to contrast and enrich data; the observations provided useful prompts for interviews, and the interviews provided an opportunity to gain an alternative or deeper understanding of what we had observed.

The study was approved by the South Central–Hampshire A Research Ethics Committee (ref 22/SC/0333). Those interested in taking part were given a participant information sheet and invited to provide consent. All participants were free to decline participation or withdraw from the study at any time. Interviews were scheduled at mutually convenient times and conducted virtually or in person at the request of the interviewee. Interpreting support was available. Interviews were digitally audio‐recorded with the consent of participants and transcribed verbatim by university‐approved transcribers and subsequently deidentified.

### 3.2 Analysis

Data analysis was undertaken alongside and after data collection. Researchers met regularly to exchange insights and questions and wrote analytical summaries with detailed contextual information and highlighted findings that they thought were especially interesting or significant. These summaries were discussed in team meetings. Researchers coded observation and interview data and worked together in a series of face‐to‐face meetings to build descriptive themes and refine our explanatory accounts. This analytical work was supported by the ‘OSOP’ (‘one sheet of paper’) method, a mind‐mapping approach to analysis that builds an understanding of the dataset by noting all issues that each section of data raises and finding connections between them (Ziebland and McPherson 2006), and charting to aid comparison across the cases (Miles et al. 2014). This paper draws heavily on a large corpus of data coded under a broad ‘reception work’ heading, which comprised data pertaining to encounters between receptionist staff and patients/family members, and observations in waiting room and back‐office areas. The focus of this paper is on the work surrounding the process of getting an appointment to see a GP. It is worth reminding the reader that reception work comprises many other tasks, such as updating records, receiving and producing letters, providing and completing forms, processing biological samples for collection and dealing with requests for prescriptions, but these are not our focus here.

The section below begins by describing how getting an appointment is accomplished. We then examine two strategies receptionists use to manage interactions with patients: *bureaucratic distancing* and *redirection.* Given the small number of practices, we have not identified which practice is referred to in the data excerpts presented.

## 4 Findings

### 4.1 Accomplishing an Appointment

The woman reaches the reception desk. She tells the receptionist that she has been contacted to make an appointment for an asthma review. The receptionist asks her name and date of birth and looks up the woman’s details on the computer. She finds her file and offers an appointment for next Tuesday at 4:25. The woman is hesitant and unsure. She asks if there is anything on a later date but earlier in the day. The receptionist offers Wednesday at 10:15 and the woman accepts this saying “Yes that is better, thanks.” The receptionist types in details, while explaining that the patient will get a text message confirming this appointment.

(Field note)

Above is a typical illustration of making an appointment at the practice reception desk, and one that Arber and Sawyer's survey respondents would probably recognise (although computer and text reminders have replaced paper‐based appointment diaries and appointment notecards). In this case, the asthma review will be conducted by a practice nurse, not a GP, and there are planned slots for these routine follow‐up activities. These details make the task of making an appointment at a time that suits the patient and the practice appear straightforward. Making an appointment to see the GP (rather than the nurse) at a future date can be accomplished in the manner outlined above but often involved additional negotiation about the date and time depending on the GP's availability. If their preferred GP was on holiday and/or in particular demand, a routine appointment might be more than 2 weeks after the request. In some cases, when all appointment slots had been filled and those further in the future were not yet available, the patient would be advised to try to book again at a future date. For those with urgent needs who wanted to be seen quickly, a same‐day ‘urgent’ appointment with a GP could be requested. One practice offered same‐day appointments on request, but at other sites these types of appointments were often more difficult to accomplish, as we show later in the paper.

Some interactions observed at the reception desk involved patients who were clearly regular visitors and thus known by name (similar to Litchfield et al.*'s* findings (2023)). Receptionists described some individual patients in warm terms (‘a sweetie’, ‘lovely’), but interviews and informal conversations also indicated that there were patients who were regarded as ‘difficult’ or ‘aggressive’. However, in contrast to studies of emergency settings (c.f. Jeffery 1979; Hughes 1989), we did not discern a systematic pattern to the offer (or denial) of appointments (e.g., based on ethnicity or age).

In addition to appointment requests made at the desk, all the practices offered phone appointment booking. Phone requests were also a feature of the system that Arber and Sawyer studied, but this route to making an appointment has become increasingly digitalised, and this has changed what is expected of patients and receptionists considerably. Practice phone systems typically operated an automated queueing system with several options for the caller, only one of which would be to speak to the appointment booking team. These phone menus helped to ‘sort’ patient calls before they reached the receptionist and allowed some queries to be directed to specific staff. For example, one receptionist might handle the urgent appointment requests, whereas another dealt with prescription requests. Digitalised phone systems typically offered eight or nine menu options, voiced to the caller in sequence, beginning, for example, with ‘emergency’ and concluding with ‘COVID vaccine request’. Listening to the full menu could take between two and 4 minutes, and in no case was ‘request a GP appointment’ the first option.

Patients often phoned as soon as the practice opened to try to secure a GP appointment. Those who had more regular contact with the practice, such as patients who had long‐term conditions or those who had been registered at the practice for several years, were often experienced in using these systems. They were well practised at phoning ‘on the dot’ at exactly 8 AM, sometimes setting multiple alarms to ensure they could phone early and get through to the receptionist. However, others spoke of difficulties getting through on the phone:

If you don’t ring at eight o’clock, the line is permanently engaged, and they’ll tell you where you are in the queue, like you could be number 17 in the queue, so you’re hanging on all that time, and of course it’s costing you money to hang on.

(Female patient aged 70+)

Patients' negotiations with receptionists about appointments could be straightforward like the asthma review described earlier or more complex, as highlighted in the examples below:

The receptionist takes a call, and says “What’s your date of birth? Ok. What do you need the appointment for?” She asks, “Since when?” and “Have you been to the pharmacist? No? Ok. Do you think you’ve been bitten? You are not sure. Ok. I can get a clinician to give you a call back. If I send you a link to your phone, would you be able to send a picture in? … I will send it to your phone. Yes … and then they will call you ….”[She closes the call, adds the patient to the call back list]

(Field note)

[Receptionist] is on the phone with a caller who would like to see doctor [GP2]. The first available appointment is nearly four weeks away. … The caller seems to not want the offered date, so the receptionist asks if they are able to do an e‐consult [online assessment]. There is more talk, and the receptionist closes the appointment booking window. Then the caller changes their mind. The receptionist reopens the booking window. The slot discussed is still available. The patient accepts the appointment.

(Field note)

The examples above highlight the use of additional digital technologies in the appointment‐making process, such as the use of a link to obtain an image and systems collectively referred to as ‘e‐consult’ (although this is the brand name of a specific assessment system) to support appointment requests. Online appointment booking via a dedicated practice website or the NHS App (which allows users to access NHS services on a smartphone or computer) was possible but not common in the practices we studied and tended to be used for appointments with allied healthcare professionals, for vaccinations, cervical smears or blood tests, rather than for GP appointments. However, all the practices offered some form of digital assessment of requests from patients or ‘e‐consultation’, and when these assessments identified that an appointment was needed, the request was automatically transferred to the reception team to review and/or phone back. Some of these appointment requests were passed to a doctor, nurse or allied health professional for a second review.

In addition to using online assessment systems, the practices also used the NHS 111 urgent care online and telephone service (Pope et al. 2022, 2017), which provided an assessment and triage process. Patients were directed to this service out of hours (when the practice was closed), and on occasion at busy periods, practices diverted callers to NHS 111 to manage workload. NHS 111 calls that completed with advice to see a GP within a specified time period required patients to return to the phone menu or practice‐based online assessment systems and, eventually, to negotiate with the receptionist to book a GP appointment.

Digitalised systems had made the process of getting an appointment more complicated. Staff and patients expressed concerns about these systems reducing access for patients who were unfamiliar with digital technology or who had difficulties communicating verbally. This led to some stereotyping, for example, claims that older people were less able to use online systems, although like Newbould et al. (2025), we found this was not always borne out in reality: interview and observational data confirmed that not all older people struggled with these systems. However, digitalisation meant that access to the GP was no longer a simple task of verbal solicitation and fulfilment (i.e., the patient asks for and the receptionist supplies an appointment). Additional work was required, by patients and staff, to navigate the many menus and options provided, and reception and back‐office staff in particular were required to manage a seemingly constant flow of digital tasks.

Arber and Sawyer's paper described a world in which the receptionist was ‘the intermediary through whom virtually all contacts between the patient and the GP are made’ (1985, 918). They argued that receptionists' use of delegated authority and discretion was the source of public hostility. Receptionists in our study also reported hostility from patients:

’We get some aggressive patients as well, that’s hard. A lot of the time they just take it out on us because we’re at the front. Some can be very irate, but more verbal, like names and things that they say like’, You’re f‐ing useless. You’re this. You’re that.’ and I just think, you’re only saying it because I’m the person that’s answered the phone’.

(Receptionist)

Staff said that when COVID‐19 cases began to decline, verbal abuse had increased; they suggested this was because patients had delayed seeking help during the pandemic and were therefore more anxious. When practices were unable to meet increased demand for appointments, patients' aggression increased. This led to staff attrition, and then new staff needed to be trained to use the complicated access systems, and response times were slower as a result. Although our data contain some reports of aggression from patients, lending support to Arber and Sawyer's analysis, we also noticed features of access interactions that led us to revisit their dragon metaphor. We identified two strategies used by reception staff to manage appointment requests that appeared to reduce hostility from patients, and one of these is closely tied to digitalisation. The next section examines how receptionists manage the problem of prioritising appointment requests before looking in more detail at these strategies.

### 4.2 Gatekeeping (Do You Really Need an Appointment With a GP?)

Arber and Sawyer argued that ‘[t]he major determinant of poor communication with receptionists is respondents' experiences of the receptionist acting in a gatekeeping role …. Patients feel antagonistic towards receptionists who are perceived as officious and interfering in medical affairs which are not seen as their legitimate province’ (1985, 918). Patients in our study also disliked this gatekeeping:

’I don’t think they should put patients in that position. They’re, kind of, saying’, Well, is it urgent? ‘Yes or no?’ and you say, ‘Well, it is urgent’, ‘Well, why is it urgent?’ ‘Well, trust me, it just is’, ‘Well, what’s wrong?’, and they want, then you’ve got to come out with, you’ll explain the condition and they’ll go, ‘Oh, yeah, I see what you mean, you better see a GP or whatever’. So, I think if you do go through the receptionist, it is not good that sometimes, they delve a little bit too deeply.

(Male patient aged 50+)

In face‐to‐face interactions at the reception desk in particular, discomfort and resulting antagonism could be pronounced due to the semi‐public location. As one older woman patient said, ‘it isn't always comfortable especially when there are people in the village that you might know’.

Arber and Sawyer were clear that not all their survey respondents experienced this interactional disclosure problem. Sometimes patients were not asked to justify their request, and some said they did not mind being asked to do so. We have examples in our data of patients disclosing symptom details at the reception desk for seemingly sensitive matters, such as breast cancer. Unlike Arber and Sawyer, we were able to ask receptionists about such interactions and watch them while they worked. We discovered that they too felt discomfort about this feature of their work:

I don’t like the way it’s gone now that the doctors are asking us to triage the [patients] and we’re not clinical, so, we can’t make that clinical decision. Obviously, we know stuff by working here but we, I, don’t like making decisions like that sometimes.

(Receptionist supervisor)

The receptionist says that some people do not want to discuss their symptoms. She finds that some questions can be quite invasive. As an example, she tells me that the other day she spoke to a woman with a cough and ended up asking her about bowel movements. This was clearly a tricky conversation.

(Field note)

Probing reasons for appointment requests made patients and reception staff feel awkward. It breached a norm that disclosure of medical information should be to a healthcare professional. This awkwardness was often accentuated by the fact that reception staff typically lived locally and may be neighbours or acquaintances of the patient they were interacting with. Digital triage could, in theory, repair this interactional breach by removing the need to disclose intimate details in person, but online systems were read and responded to by receptionists and funnelled patients to speak to a receptionist, at which point symptom details and urgency were revisited in person, with accompanying disclosure discomfort.

Our analysis suggests that interactional discomfort and potential antagonism continue to surround the receptionists' gatekeeper role. However, as we show in the next section, receptionists have developed two strategies that can mitigate the potential for hostility from patients.

### 4.3 Bureaucratic Distancing

The computerised appointment systems used in our case study practices embargoed appointments for future release, in the expectation that the next day or week would inevitably produce a new set of requests for appointments. The cap on the number of appointments available each day meant that available slots were often rapidly filled. At busy times (Monday mornings, Fridays and days preceding or after bank holidays when the practice would be closed), appointments could run out within 10–15 min. This was especially frustrating to those patients who waited on the phone only to find that all the appointments were gone when they reached the front of the queue.

The first requests for an appointment (by phone or in person) each day were typically concise conversations and often resulted in an appointment booking. Some appointment slots were held back (embargoed) ‘for emergencies’ and were especially closely guarded by the reception staff. Some of these slots would be filled after GPs or other clinical staff conducted phone consultations and call backs (identified by sifting the online requests), and some were protected for use by patients deemed ‘special’, such as children, the very elderly, those who were at the end of life or those who had mental health conditions. As the day went on, appointment scarcity increased and receptionists defended available slots more carefully, often using the term ‘emergency’ rather than ‘urgent’ in conversations with patients and seeking more confirmation that the need was especially pressing. Patients were aware of this, as in this example:

There was one particular lady, I don’t know if she’s still there. She is quite abrupt. (Interviewer: *could you describe what you mean by that?*) She’ll be like, ‘It’s not an, an emergency, there’s only one doctor on. You’ll have to, what do you call it, ‘ring back tomorrow’.

(Female patient aged 30+)

After negative media coverage and policy guidance, receptionists were heavily discouraged from saying ‘call again tomorrow’, though in our observations many reception staff resorted to this advice when all the slots available were full (especially in the one practice that only offered same‐day appointments). However, they also used other conversational tactics: Frequent phrases included ‘it all depends on appointments being given to us’ and ‘there's nothing left on the system’. This talk conveyed a passive sense of resignation:

The receptionist is struggling to make appointment for an elderly man. She repeats to the patient again that she needs to find out the duty doctor’s availability first. After several goes at looking up appointments on the computer, pressing buttons on the keyboard, and looking frustrated, the elderly man finally understands, says “Alright” and sits down to wait. A little later she comes over and says, apologetically, to the man “All the appointments we’ve got today have gone.”

(Field note)

Use of these passive phrases helped to imply that the receptionist had somehow joined the patient in their quest to obtain an appointment. The receptionist, far from being a dragon, was recast as a powerless digital slave; she too was thwarted by a faceless, digital system. She was ‘on their side’. This repositioning was emphasised by tapping the keyboard, staring at the computer screen waiting for the diary to refresh and expressing audible surprise or apparent sadness, for example by sighing, at the lack of available appointments. One member of staff (referring colloquially to the receptionists as ‘the girls’) described this ‘flicking’ between screens on the computer:

The more experienced girls, the senior girls that have been here, they’re constantly flicking like, ‘Yes, that’s …. I can’t find an appointment’, and then the actual supervisor’s looking, flicking through, she said, ‘I can’t find one’, and she was trying really hard.

(Receptionist)

We labelled this strategy of locating the problem of access elsewhere as *bureaucratic distancing.* Bureaucratic distancing managed the interactional difficulty of having limited appointments and fostered allyship between receptionists and patients. This strategy was entwined with and made possible by the digitalisation of general practice. The performance of ‘searching’ for an appointment on a computer (whether in person or on the phone) moved the locus of rationing ‘into the machine’ and cleverly conveyed the sense that receptionists were aligned with patients in the difficult task of extracting an appointment from ‘the system’. Bureaucratic distancing allowed performative displays of effort by the receptionist. She could show that she was ‘trying hard’ to find an appointment. Sometimes she was ‘lucky’ in her quest or on rarer occasions might ‘squeeze in’ or ‘find’ an appointment, extending her allyship further.

Bureaucratic distancing moved the problem of scarcity of appointments outside the receptionist‐patient orbit. Where Arber and Sawyer suggested that the receptionist had become a visible barrier between the patient and the GP, this strategic move positioned the source of the trouble elsewhere, inside the computer or in the wider health system. As an interactional strategy, bureaucratic distancing helped to diminish the likelihood of a hostile response from the patient. It made receptionists appear less dragon‐like and reduced the likelihood that hostility would be directed at them. Our data include surprisingly numerous examples of patients describing reception staff as ‘friendly’ and ‘really nice’ people. On occasion, we witnessed patients joining in with the performance and sighing along with the receptionist at the lack of appointments. Some patients empathised with the difficulty of the access task:

I do have to say, the receptionists [are] very, very good. They are good. It’s just one of those [things] where they’re up against it. Everybody seems to be up against it.

(Male patient aged 70+)

Bureaucratic distancing was an important interactional strategy deployed to good effect by reception staff to manage GP access and reduce potential hostility from patients. The next section explores a second strategy, *redirection,* that receptionists also used.

### 4.4 Redirection (Can We Offer You Something Else Instead?)

Another way receptionists managed the interactional difficulty of not being able to offer a GP appointment was to divert or *redirect* appointment requests to a non‐GP healthcare provider. This could include suggesting that the patient visit an urgent care centre (an alternative to hospital emergency department care that provides care for injuries and illnesses that require prompt attention but are not life‐threatening) or a local community pharmacy (where over‐the‐counter medicines for common ailments can be obtained along with health advice). Within the practice, there were additional sources of advice and care including paramedics, nurses and physiotherapists. Diverting requests to these alternatives provided an immediate solution to the receptionists' interactional problem: patients were removed from the queue for a GP appointment and were often placated (even if temporarily).

The [receptionist] said, ‘Oh, well, we can’t get you anything for a month’. So, I said, ‘Oh, okay. What, what’s the alternatives?’ So, then they, they said, ‘Here’s a piece of paper with all the different places you can go’.

(Female patient aged 50+)

One staff member described the utility of such redirection as follows:

It makes you feel a little bit more relaxed if you’re starting the day knowing that there’s, we’ve got all these appointments. … you feel a bit more positive, that we have got something to offer the patients …. Cause we don’t, as I said earlier, we don’t like to say no.

(Receptionist/healthcare assistant)

However, as an interactional strategy, redirection might only work in the short term. The Pharmacy First scheme had encouraged redirection of minor illnesses and symptoms to community pharmacists. Sometimes the community pharmacy was located next to the practice or in the same building. Patients were advised to see the pharmacist as an alternative source of care, but patients and staff described many instances when pharmacists referred patients back to the GP, sometimes on the same day. In practices that employed physiotherapists and/or paramedics, certain conditions (such as musculoskeletal problems or minor injuries such as sprains) could be directed to them instead of the GP. However, as with pharmacists, sometimes these cases reappeared as requests for a GP appointment when the practitioner concerned found that their ‘scope of practice’ or knowledge did not allow them to treat the presenting complaint. This led to some scepticism from clinical and reception staff about non‐GP roles and the utility of these alternative sources of care. Patients too complained of being ‘fobbed off’ or ‘going round the houses’ (phrases used to convey circularity and frustration associated with being diverted to forms of care that, they felt, were less expert than the GP).

Nonetheless, as a strategy, redirection gave receptionists ‘something to offer patients’ instead of a GP appointment. As this practice manager explained, directing patients to online assessment forms was one such strategy:

I think the e‐consults help and I am at times quite stubborn with clinicians about the use of e‐consults because it allows us, it gives the staff an opportunity to give the patient something.

Redirection was supported by digitalisation and this provision of online assessment. It was also reinforced by national policy, notably the Additional Roles Reimbursement Scheme (NHS England 2023), which provided reimbursement for the salaries of non‐GP roles. Much was made of the idea of ‘signposting’ patients to the ‘right service for them’ and directing them to the ‘most appropriate’ care provider. This rhetoric legitimated and bolstered the use of redirection as a strategy by reception staff. In addition, sending the patient to an alternative provider ‘worked’ in the moment; it offered something rather than nothing and could, albeit temporarily, dissipate potential hostility from patients.

These two strategies of bureaucratic distancing and redirection were successfully deployed to manage the interactional difficulties reception staff encountered when managing access to the GP. The next section discusses these findings.

## 5 Discussion and Conclusion

NHS activity data reported that 43.2% of appointments in September 2024 took place on the same day that they were booked and 44% of all appointments were carried out by a GP (NHS England Digital 2024). Many patients do obtain an appointment with a GP, and often this will be a consultation on the same day (by phone or in person), or, more typically, as in most of our sites, a doctor or nurse telephone call back that might lead to this appointment. The British Medical Association (2024) has described practices offering ‘record breaking’ numbers of appointments. Nonetheless, as per Arber and Sawyer's research in 1985, more recent commentary by Tonkin (2022) and the review by Willer et al. (2023), the perceived scarcity of appointments and the digital and human systems used to manage demand continue to fuel hostility and verbal and physical violence towards receptionists and practice staff. Ward and McMurray's work (2011) showed how receptionists deploy emotional neutrality in response to abuse from patients and persuasively argued that receptionists engage in interactional performances intended to produce a particular frame of mind in patients. Our analysis builds on this earlier work and shows that receptionists have added to this strategic repertoire by using bureaucratic distancing and redirection to dissipate patients' potential anger and frustration.

Some of the tasks performed by reception staff have not changed since Arber and Sawyer's study. They argued that reception staff in general practice had substantial informal, but little formal, power (1985, 912), were formidable as gatekeepers to GP access and provoked considerable hostility from patients. We have shown that receptionists still ration appointments and establish the legitimacy of access requests, particularly when dealing with urgent and same‐day requests. Our data confirm that the need for medical disclosures to secure an appointment is sometimes felt by patients as interactionally awkward, and we found that receptionists often feel this discomfort too. This can be a source of hostility. However, we have also shown that often patients and receptionists appear allied, or united in the quest to obtain an appointment, particularly in relation to digitalised systems.

Appointment‐making is still accomplished by phone or at the reception desk, but, as we have shown, it is increasingly mediated by digital technologies. We agree with Stockwell et al. (2024) that the nature of demand management performed by receptionists is more complex. Digitalisation has added to this complexity but has also offered a valuable strategic opportunity, namely, bureaucratic distancing. Digitalisation has configured the receptionist as an ally of the patient. She is no longer a dragon at the desk, but instead, like them, she can be positioned as a powerless slave of a digital system. The computer says no. The appointments have run out. She cannot be blamed.

Alongside bureaucratic distancing, receptionists have a second strategy to counteract hostility when patients seek an appointment with a GP. They can redirect patients to a different care provider. Along with digitalisation, this is a key change in general practice since Arber and Sawyer wrote their paper. Although policy has not delivered substantial increases in the supply of GPs (despite promises by successive governments; see, for example, Iacobucci 2019), there have been significant attempts to enrol other healthcare professionals and nonclinical staff in delivering care. The rise in numbers of these other staff has not necessarily delivered the access solution preferred by patients, who in many instances would rather see their GP, but it has provided a valuable strategic device for reception staff. The offer of an appointment with a nurse or a paramedic or the suggestion that ‘the pharmacist may help’ allows receptionists to redirect requests for GP appointments. By offering or suggesting alternatives to a GP appointment, she enacts health policy and softens the denial of a GP appointment. As we observed, the effectiveness of this strategy can be short‐lived. Moreover, as Checkland et al. (2025) have noted, keeping up with the proliferation of these additional roles and understanding what they can offer to patients has added to the complexity of receptionists’ work.

We suggest that the interactional strategies of bureaucratic distancing and redirection provide a kind of glue that unites the patient and the receptionist in the GP‐receptionist‐patient triad. Previous sociological scholarship in emergency department settings (e.g., Jeffery 1979; Hughes 1989; Hillman 2014) demonstrated that certain types of patients may be stereotyped and treated differently. In our data, although a small number of patients were given priority based largely on illness presentation (e.g., end of life, mental illness) or age (very young or very old), it appeared that as appointment slots decreased as the day progressed, bureaucratic distancing and redirection, along with its associated performative effort, was displayed to all comers.

In proposing the concept of bureaucratic distancing, our analysis augments ideas about the invisible work implicated in digitalisation (Bergey et al. 2019; Nicolini 2006; Trupia et al. 2021) and provides further evidence about the ways that information technologies reconfigure health and other forms of work. In the context of UK healthcare, these observations about digitalisation are especially important given the direction of health policy. The Darzi report (Darzi 2008) and the recent ‘Fit for the future: 10 Year Health Plan for England’ announced by the Labour government in July 2025 have embraced and emphasised the ‘shift from analogue to digital’ (UK Government 2025). The UK Secretary of State for Health and Social Care announced an end to ‘the 8AM scramble’ and that people ‘who need one will be able to get a same day GP appointment’. This promise is founded on increasing the use of digital technologies, notably the NHS App for appointment booking. This App has been described anthropomorphically as a ‘doctor in your pocket’ but offers another form of bureaucratic distancing and crucially does nothing to increase the supply of GP appointments.

Our data come from eight English case studies and were collected as the UK NHS was readjusting to face‐to‐face GP provision post‐pandemic. There will undoubtedly have been further changes to appointment systems since we conducted our fieldwork, and there are features of access systems elsewhere that we have not examined, for example, slightly different phone menus or online assessment systems, or local variation in how receptionists manage access. Nonetheless, our research has provided sufficient thick description to suggest that our core findings are transferable to similar NHS general practice settings and potentially to other settings where street‐level bureaucrats attempt to manage scarcity of supply.

Patients present to general practice seeking an appointment with a GP, but the barrier to getting an appointment is no longer solely the human receptionist. Patients on a ‘quest’ to obtain ‘the prize’ of a GP appointment may still be thwarted and sometimes meet dragons at the desk, but they may more often find themselves allied with the receptionist. Receptionists’ strategies help to reduce hostility, but the problem of GP access remains. Bureaucratic distancing and redirection to other care providers can assist with managing demand on a daily basis, but ultimately the bugbear is the access system, digitalisation and alternative care provision. The problem of GP access will persist unless the problem of supply, namely, the availability of appointments with the GP, is addressed.

## Author Contributions

**Catherine Pope:** conceptualization (equal), data curation (equal), formal analysis (equal), funding acquisition (equal), investigation (equal), writing – original draft (lead). **Annelieke Driessen:** data curation (equal), formal analysis (equal), investigation (equal), writing – review and editing (supporting). **Abi Eccles:** data curation (equal), formal analysis (equal), investigation (equal), writing – review and editing (supporting). **Bella Wheeler:** data curation (supporting), formal analysis (supporting), writing – review and editing (supporting). **Carol Bryce:** data curation (equal), formal analysis (equal), investigation (equal), writing – review and editing (supporting). **Jacob Heath:** data curation (supporting), formal analysis (supporting), investigation (supporting), writing – review and editing (supporting). **Chloe Phillips:** data curation (supporting), formal analysis (supporting), writing – review and editing (supporting). **Toto Gronlund:** data curation (supporting), formal analysis (supporting), writing – review and editing (supporting). **Helen Atherton:** conceptualization (equal), data curation (equal), formal analysis (equal), funding acquisition (equal), investigation (equal), supervision (equal), writing – review and editing (supporting).

## Ethics Statement

This study received ethical approval from South Central–Hampshire A Research Ethics Committee ref 22/SC/0333.

## Consent

The authors have nothing to report.

## Conflicts of Interest

The authors declare no conflicts of interest.

## Permission to Reproduce Material From Other Sources

The authors have nothing to report.

## Data Availability

The data are not publicly available due to privacy or ethical restrictions.
